# Aramid Nanomaterials of Various Morphologies: Preparation and Mechanical Property Enhancement

**DOI:** 10.3389/fchem.2019.00939

**Published:** 2020-01-17

**Authors:** Congcong Dong, Peng Guo, Yue Yuan, Changmei Sun, Rongjun Qu, Chunnuan Ji, Ying Zhang, Ying Wang

**Affiliations:** School of Chemistry and Materials Science, Ludong University, Yantai, China

**Keywords:** aramid nanofibers, alkylation, morphology, PVC, mechanical properties

## Abstract

Aramid nanofibers (ANFs) are a novel type of promising nanoscale building blocks for high-performance nanocomposites. Conventionally, ANFs are used to composite with polymers containing polar groups such as –OH and –NH_2_ since those polymers can interact with the amide groups in ANFs through polar-polar interaction such as hydrogen bonding. In this study, ANFs were derivatized with non-polar alkyl groups including ethyl, octyl and dodecyl groups and used as a performance-enhancing additive to polyvinyl chloride (PVC) with weak polarity. Interestingly, it was observed that the morphologies of the resulting alkyl-derivatized aramid nanomaterials (R-ANMs) varied significantly including nanofibers, nanobranches, nanosheets, and nanospheres, all of which depended on the degree of substitution (*DS*) and the chain length of the alkyl group. As an additive, R-ANMs improved the Young's modulus, toughness and yield strength of the PVC films. This study proves the concept that ANFs can be used to composite weakly polar or non-polar polymers.

## Introduction

Poly(p-phenylene terephthalamide) (PPTA), known by its trade name Kevlar, is a type of aromatic polyamide (aramid) material with high strength, stiffness and thermal stability (Tanner et al., 1989; O'Connor et al., 2008, 2009a). Due to these desirable properties, aramid fibers, particularly in the form of microfibers or nanofibers, are widely used for reinforcing composite materials (Mukherjee et al., 2006; Reis et al., 2012). However, it can be challenging to select an appropriate preparation method for aramid fiber reinforced composite. First of all, direct blending has shown to cause phase separation and thus limited mechanical property enhancement as a result of the poor adhesion between aramid fibers and the polymer matrix (Leal et al., 2009; Chen et al., 2012; Sa et al., 2014). Moreover, nanofiber preparation methods such as drawing (Ondarcuhu and Joachim, 1998), templated synthesis (Feng et al., 2002), and electro-spinning (Ramakrishna et al., 2006), are not suitable for aramid nanofibers (ANFs) owing to the inherent inertness of aramid (Ifuku et al., 2014).

Recently, negatively charged, uniformly sized ANFs were successfully prepared in DMSO as a stable dispersion by controlled deprotonation with KOH (Yang et al., 2011). Such dispersion represents the first example of nanofiber dispersion of synthetic polymers. Since then, several ANF-reinforced composites have been reported, such as ANFs/polyethylene glycol (PEG) nanocomposite films (Tung et al., 2015), ANFs/polylactic acid (PAA) nanocomposite films (Yang et al., 2015), ANFs/polyurethane (PU) composites (Kuang et al., 2015), ANFs/polyvinyl alcohol (PVA) (Guan et al., 2017) and epoxy resin (Lin et al., 2017). ANFs greatly improved the mechanical strength of the resulting composites as they are considered new nanoscale building blocks for ultrastrong materials. For example, the ANF-enhanced PU showed a record modulus of 5.275 GPa and a breaking strength of 98.02 MPa. The polymer matrices of ANF-reinforced composites, as exemplified by the examples above, mostly contain functional groups as –OH and –NH_2_ that can interact with –CONH- groups in ANFs through hydrogen bonds, ionic interaction or dipole interaction. Reports of ANF-reinforced composites based on a polymer matrix lacking functionalities, e.g., PE, PP, PVC, are relatively scarce.

Recent success of using ANFs modified carbon nanotubes as PVC additives (Pan et al., 2017; Fu et al., 2019) inspired us to explore the possibility of using ANFs derivatized with non-polar groups as PVC additives. With direct derivatization via N-substitution, a less polar aramid nanomaterial should have better solubility in common solvent, enhanced miscibility in the polymer composite, and decreased melting point (Biggs et al., 1946).

In this paper, ANFs were derivatized with ethyl, octyl and dodecyl groups via N-substitution. The morphologies of the resulting R-ANMs were studied in relation to the alkyl group chain length and the degree of substitution. A comprehensive investigation on their performance-enhancing properties in PVC was also conducted.

## Experimental

### Materials

Bulk Kevlar 964C was provided by DuPont. Dimethylsulfoxide (DMSO) was purchased from Kishida Chemicals (Tokyo, Japan). DMSO was dried with calcium hydride and distilled prior to use. PVC with *K* value of 59–55 was obtained from Aladdin Chemistry Co. Ltd. KOH were purchased from Sinopharm Chemical Reagent Co Ltd (SCRC) and were used without further purification. Reagent-grade Bromoethane, 1-Bromooctane, and 1-Bromododecane were obtained from Jiuding Chemistry and were used as received. The purities of the reagents ranged between 90 and 99%. Water used in this study was de-ionized water.

### Instrumentation

Elemental analysis was performed on an Elementar VarioEL III instrument, Elementar Co, Germany for the determination of carbon, nitrogen and hydrogen contents. FTIR spectra were collected in the wavenumber range of 700–4,000 cm^−1^ with 64 scans at a 2 cm^−1^ resolution. The microstructures of the R-ANMs were studied by transmission electron microscopy (TEM, Hitachi H-800, Japan). Nitrogen was used as the purge gas. The XRD patterns of samples were measured with a Shimadzu LabX XRD-6100 diffractometer using CuKa radiation. Thermogravimetric analysis (TGA) was performed on a PerkinElmer TGA 2050 instrument under nitrogen. The heating rate for the polymer composite samples was 10–30 K min^−1^. Optical microscopic images of the composite films were captured using an Olympus GX51. Tensile measurements of the R-ANMs/PVC films were taken with an Instron 4465 instrument equipped with a 5 KN load cell under the ambient conditions and each sample was tested at a crosshead speed of 10 mm/min. All of the samples were cut into strips with an effective gauge length, width, and thickness were 30, 10, and 0.26 mm, respectively. Five separate strips of each composite film sample were measured. The reported values were calculated as averages over three specimens for each group of specimens.

### Preparation of ANFs/DMSO Dispersion

**Figure 1:**
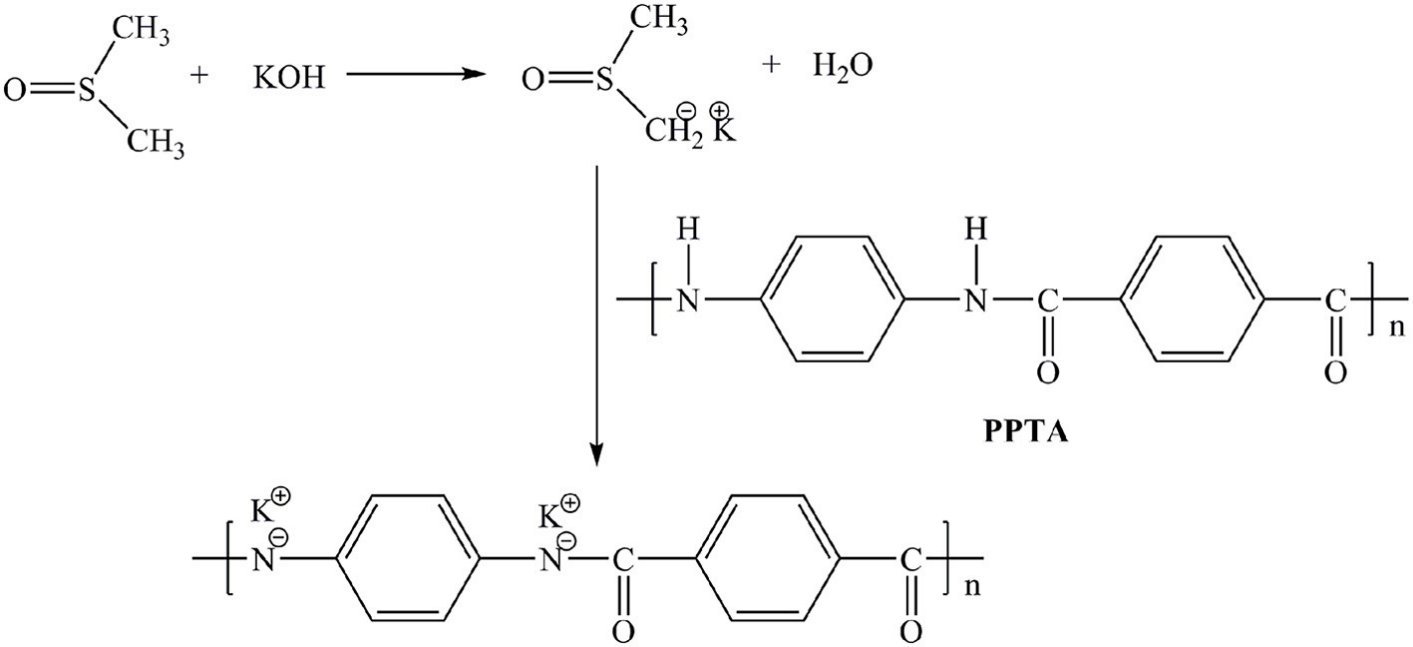
Synthetic scheme of ANFs/DMSO dispersion.

ANFs/DMSO dispersion was prepared by splitting the bulk Kevlar 964C threads in DMSO with the aid of KOH as previously reported (Yang et al., 2011). Typically, 0.6 g of bulk Kevlar 964C threads and 0.9 g of KOH were added into 300 mL of DMSO. The mixture was stirred at room temperature for 1 week and a dark red ANFs/DMSO dispersion was obtained. The metalation reaction is presented in Figure 1.

### Synthesis of R-ANMs

**Figure 2:**
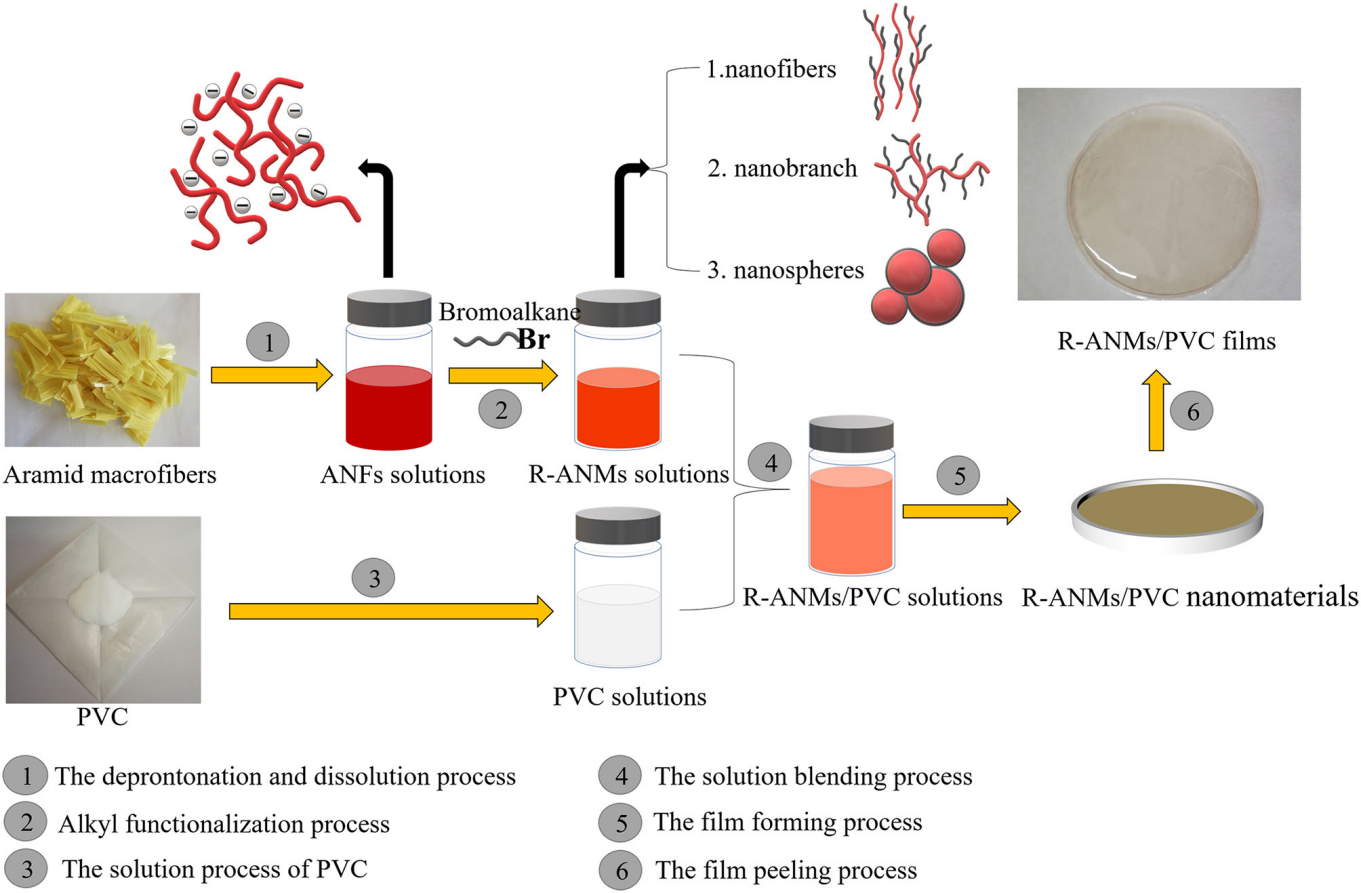
Schematic illustration of the fabrication process of R-ANMs/PVC films.

ANMs-C_2_H_5_-X samples with different degrees of ethyl substitution were prepared according to the methods described in Takayanagi and Katayose (1981). Sample notations and their corresponding reactant compositions are shown in Table 1. In a typical procedure for ANMs-C_2_H_5_-1, 50 mL of ANFs were added to a flask protected by nitrogen. Then, 4.58 mg of BrC_2_H_5_ was added and the mixture was stirred at 30°C for 16 h. The polymer was precipitated in a large excess of water, filtered, washed several times with water, acetone and ethanol, and then dried at 80°C for 5 h in a vacuum oven. ANMs-C_8_H_17_-X and ANMs-C_12_H_25_-X were prepared similarly using BrC_8_H_17_ and BrC_12_H_25_ instead of BrC_2_H_5_. The synthesis diagram of alkyl functionalized ANFs is shown in Figure 2. Elemental composition data from the elemental analysis are also given in Table 1.

**Table 1:** Synthetic formulation, elemental composition, and degree of substitution of the R-ANMs samples.

**Sample notation**	**Alkylation reagent**	**ANFs (mL)**	**Alkylation reagent (mg)**	**Elemental composition (%)**			**Degree of substitution (%)**
				**N**	**C**	**H**	
ANMs-C_2_H_5_-1		50	4.58	10.70	66.27	4.52	11.29
ANMs-C_2_H_5_-2		50	9.16	13.52	87.26	6.98	23.23
ANMs-C_2_H_5_-3	Bromoethane	50	18.31	10.19	66.2	5.34	33.64
ANMs-C_2_H_5_-4		50	36.63	9.61	64.26	5.42	40.06
ANMs-C_2_H_5_-5		50	91.57	7.53	51.67	4.32	50.28
ANMs-C_2_H_5_-6		50	183.14	9.56	70.43	6.32	79.75
ANMs-C_8_H_17_-1		50	8.11	11.13	70.73	5.20	5.48
ANMs-C_8_H_17_-2		50	16.23	9.77	67.49	5.563	13.24
ANMs-C_8_H_17_-3	Bromooctane	50	32.44	9.83	73.58	6.21	21.66
ANMs-C_8_H_17_-4		50	64.91	9.22	74.21	6.72	29.88
ANMs-C_8_H_17_-5		50	162.29	7.04	59.2	5.51	35.13
ANMs-C_8_H_17_-6		50	324.57	7.32	79.09	8.74	70.07
ANMs-C_12_H_25_-1		50	10.47	11.43	72.02	5.02	2.93
ANMs-C_12_H_25_-2		50	20.94	7.94	56.95	4.48	11.40
ANMs-C_12_H_25_-3	1-Bromododecane	50	41.88	7.16	58.43	5.23	21.01
ANMs-C_12_H_25_-4		50	83.76	8.27	75.28	7.51	30.17
ANMs-C_12_H_25_-5		50	209.44	7.1	74.09	8.04	43.12
ANMs-C_12_H_25_-6		50	418.87	4.3	58.5	7.29	73.93

### Fabrication of R-ANMs/PVC Nanocomposite Films

R-ANMs/PVC nanocomposite films were prepared by a simple solution casting method (Figure 2). First, a 13.33 wt% PVC solution in DMF was prepared by stirring 2 g PVC in 15 mL DMF at 70°C for 1 h (Figure 2③). A certain amount of R-ANMs/DMSO dispersion (0.6 mg/mL) was then gradually added into the PVC solution under continuous stirring at 70°C for 10 min. The obtained dispersions were sonicated for another 15 min to remove air bubbles (Figure 2④). After sonication, the homogeneous and uniform dispersion was cast onto a horizontal plate with a diameter of 9 cm and dried at 60°C for 48 h (Figure 2⑤). The thickness of those films was measured to be 0.2–0.3 mm (Figure 2⑥). The R-ANMs/PVC films were denoted as ANMs-C_2_H_5_-X/PVC, ANMs-C_8_H_17_-X/PVC, and ANMs-C_12_H_25_-X/PVC.

## Results and Discussion

### Preparation of ANFs/DMSO Dispersion

**Figure 3:**
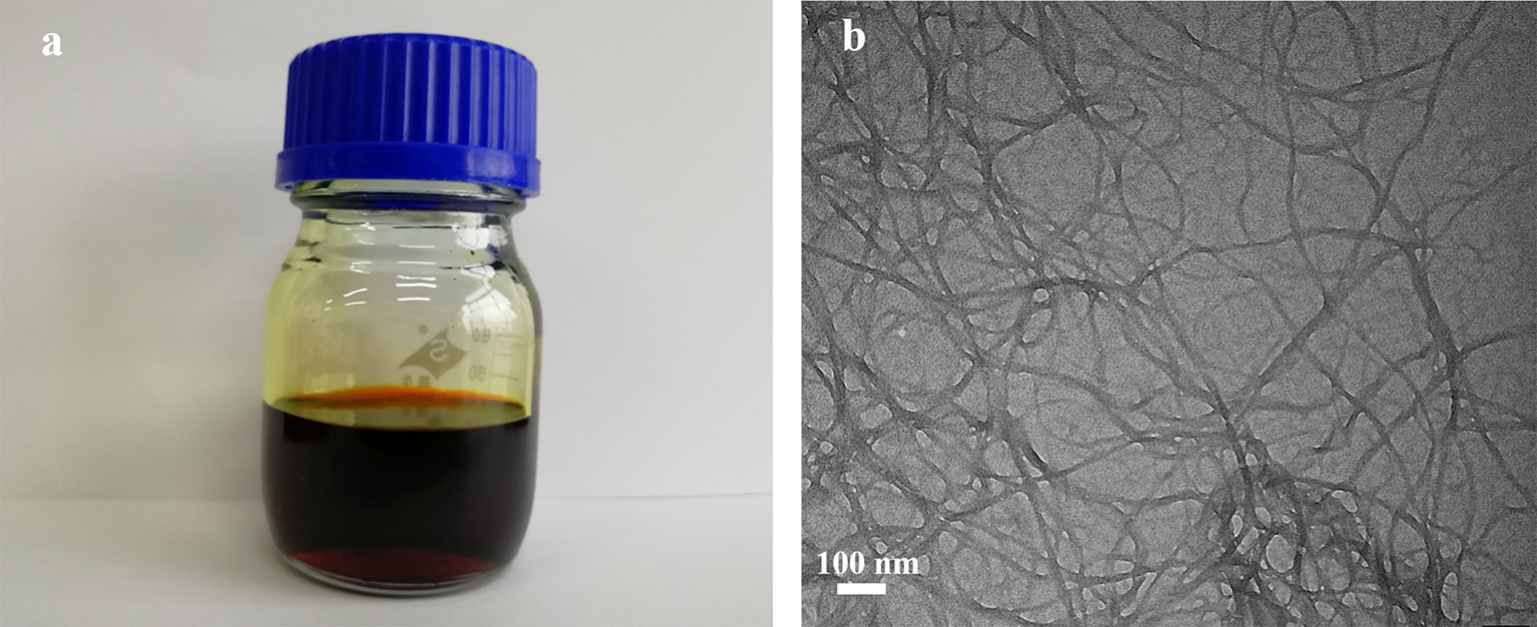
**(a)** Photograph of ANFs/DMSO dispersion **(b)** TEM image of ANFs.

The Kevlar-964C threads can be effectively split into aramid nanofibers by deprotonation in the solution system of DMSO and KOH according to the literature (Yang et al., 2011). The mechanism is likely through abstraction of mobile hydrogen from amide groups and substantial reduction of hydrogen bonds between polymer chains, resulting in the formation of negatively charged nitrogen ions (Takayanagi et al., 1982; Burch et al., 1990; Burch and Manring, 1991). The electrostatic repulsion between the polymer chains facilitates the formation of stable homogeneous dispersion. This mechanism is similar to that of the stable suspensions of negatively charged GO. However, the degree of disintegration for ANFs was limited and did not reach the molecular level due to π-π stacking in the polymer chains (Fan et al., 2012). As shown in Figure 3a, the ANFs/DMSO dispersion is red, homogeneous and stable for several months. The morphologies of ANFs were characterized by TEM. The estimated diameter of the prepared ANFs from the TEM image (Figure 3b) was approximately 25 nm, which is in consistent with the literature whose the length of ANFs appears to be in the range of 5–10 μm and the diameters of ANFs after the dissolution of Kevlar fabric are 20–30 nm (Yang et al., 2011).

### Characterization of ANMs-C_2_H_5_-X, ANMs-C_8_H_17_-X, and ANMs-C_12_H_25_-X

#### FTIR Analysis

**Figure 4:**
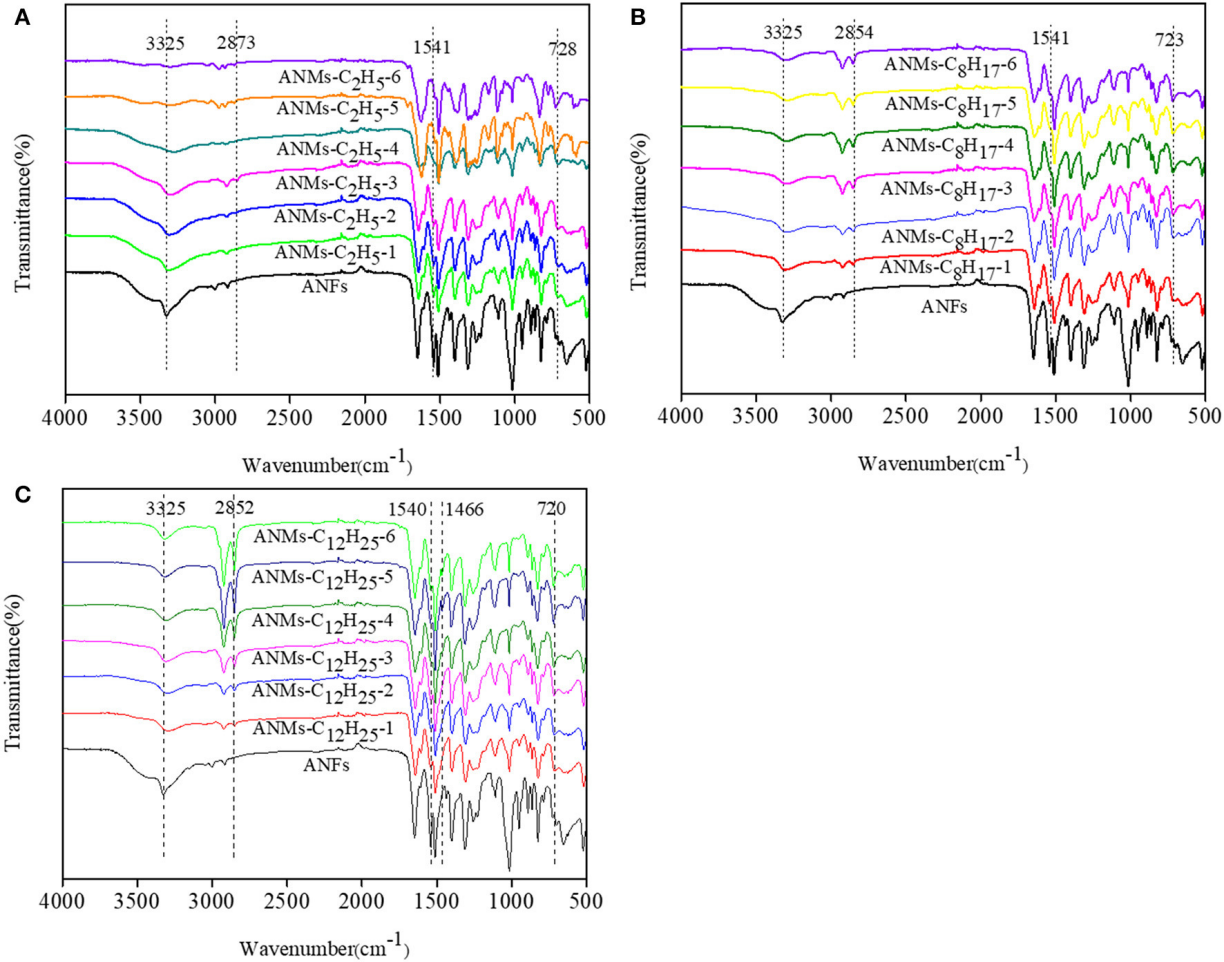
IR spectra of **(A)** ANFs, and ANMs-C_2_H_5_-X, **(B)** ANFs, and ANMs-C_8_H_17_-X, **(C)** ANFs, and ANMs-C_12_H_25_-X.

FTIR spectra of ANFs, ANMs-C_2_H_5_-X, ANMs-C_8_H_17_-X and ANMs-C_12_H_25_-X are shown in Figure 4. The broad peak at about 3,325 cm^−1^ and the narrow peak at about 1,541 cm^−1^, which can be attributed to the stretching of N–H (Takayanagi and Katayose, 1981) and the flexural and telescopic vibration of -NH in ANFs, respectively, are significantly weakened in the spectra of R-ANMs as compared to those in the spectrum of ANFs. The extent of decrease is also positively correlated to the degree of substitution. This indicates that substitution occurred at the –N-H site. Meanwhile, a strong absorption peak at about 2,873 cm^−1^, which can be attributed to -CH_2_- stretching in aliphatic group, increases with the degree of substitution in R-ANMs. The characteristic peak of alkyl at about 723 cm^−1^ appears in the spectra of all R-ANMs as compared to ANFs. These two new peaks suggest the successful introduction of alkyl group onto ANFs. For ANMs-C_12_H_25_-X samples, there is a new absorption peak at around 1,466 cm^−1^, which can be attributed to the methylene groups on the side chains of dodecyl groups. In sum, it is suggested in the FTIR spectra that alkyl derivatization was successful at the N-H site when R-ANMs were synthesized from ANFs.

#### XRD Analysis

**Figure 5:**
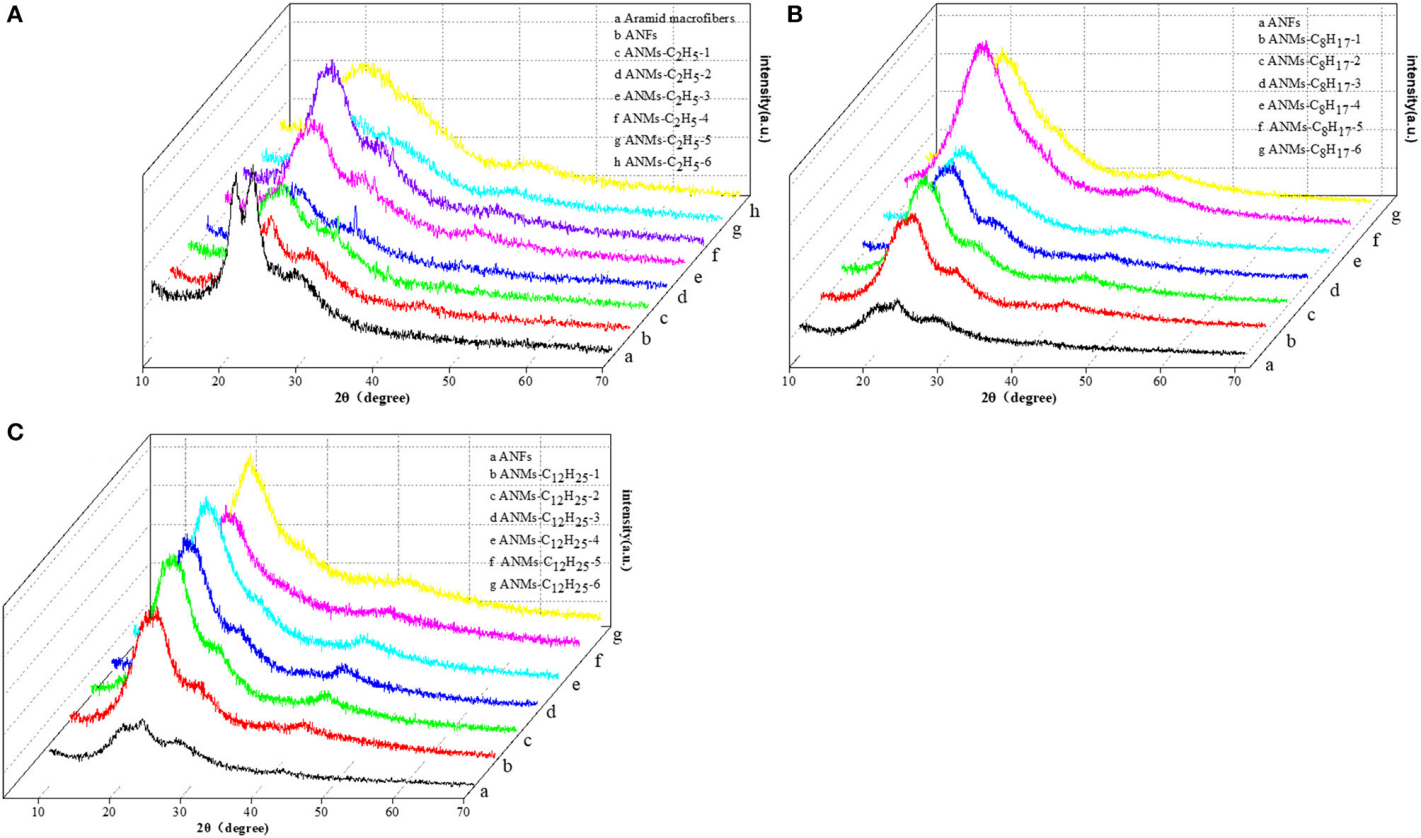
XRD patterns of **(A)** PPTA, ANFs, and ANMs-C_2_H_5_-X, **(B)** ANFs and ANMs-C_8_H_17_-X, **(C)** ANFs and ANMs-C_12_H_25_-X.

The wide angle x-ray diffraction patterns of the powdered ANFs, ANMs-C_2_H_5_-X, ANMs-C_8_H_17_-X, and ANMs-C_12_H_25_-X at room temperature are overlaid in Figure 5. Three characteristic peaks are apparent in both macroaramid and aramid nanofibers. The 2θ angles of 20°, 23°, and 28° correspond to the reflections of (110), (200), and (004) planes, respectively (Northolt, 1974). It is known that PPTA molecules are axially oriented in Kevlar fibers (aramid macrofibers). The ANFs show almost the same state of PPTA orientation, revealing that the PPTA molecules are highly aggregated and orientated (Yan et al., 2016). It also indicates that ANFs still retain to a large extent the crystallization of aramid macrofibers, inheriting the mechanical properties of the macrofibers.

With the increasing degree of derivatization for ANMs-C_2_H_5_-X samples, as show in Figure 5A, the three peaks broaden, accompanied by the merger of the two peaks around 20° and a shift to low angle. At the same time, the (004) peak at around 28° weakens and eventually disappears. This peak corresponds to the regularity of PPTA along the molecular axis and indicates the existence of a stiff chain sequence which is effective for reinforcement of flexible coil-like chains. At low degree of derivatization, ANMs-C_2_H_5_-X still retains the stiff chain sequences expected to be effective in reinforcement. At high degree of derivatization, the polymers lose the intramolecular regularity, which accompanies the loss of rigidity and results in an amorphous state. As the number of carbon atoms in the alkyl radical increases, For ANMs-C_8_H_17_-X and ANMs-C_12_H_25_-X samples with longer carbon chains (Figures 5B,C), there appears a broad peak of medium intensity centered at about 21°. Overall, the XRD patterns show that the derivatization reaction by bromoalkanes was successful and that the crystallinity of alkyl modified ANFs decreases with the increasing degree of substitution.

#### TEM Analysis

**Figure 6:**
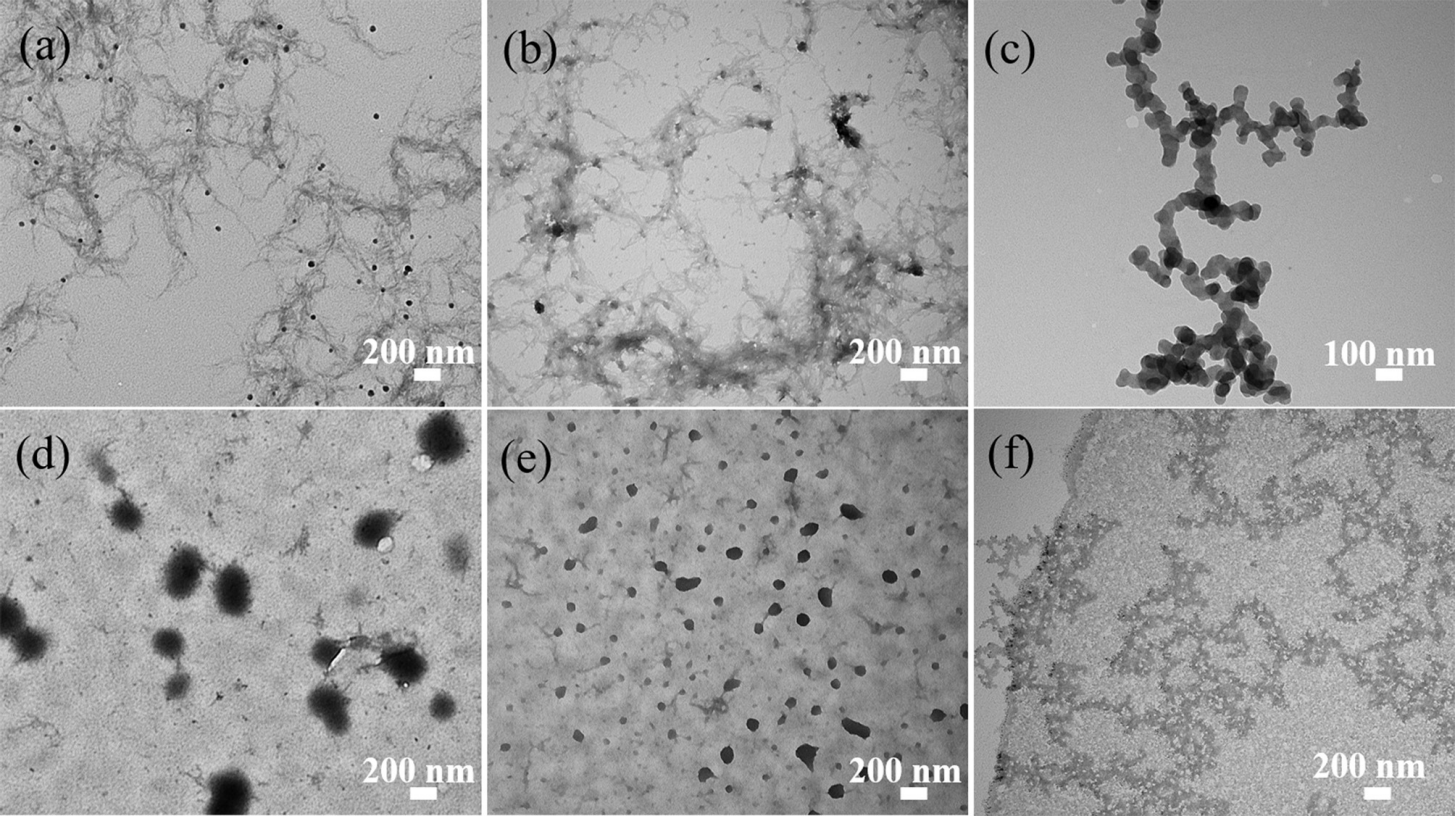
TEM images of **(a)** ANMs-C_2_H_5_-1, **(b)** ANMs-C_2_H_5_-2, **(c)** ANMs-C_2_H_5_-3, **(d)** ANMs-C_2_H_5_-4, **(e)** ANMs-C_2_H_5_-5, **(f)** ANMs-C_2_H_5_-6.

**Figure 7:**
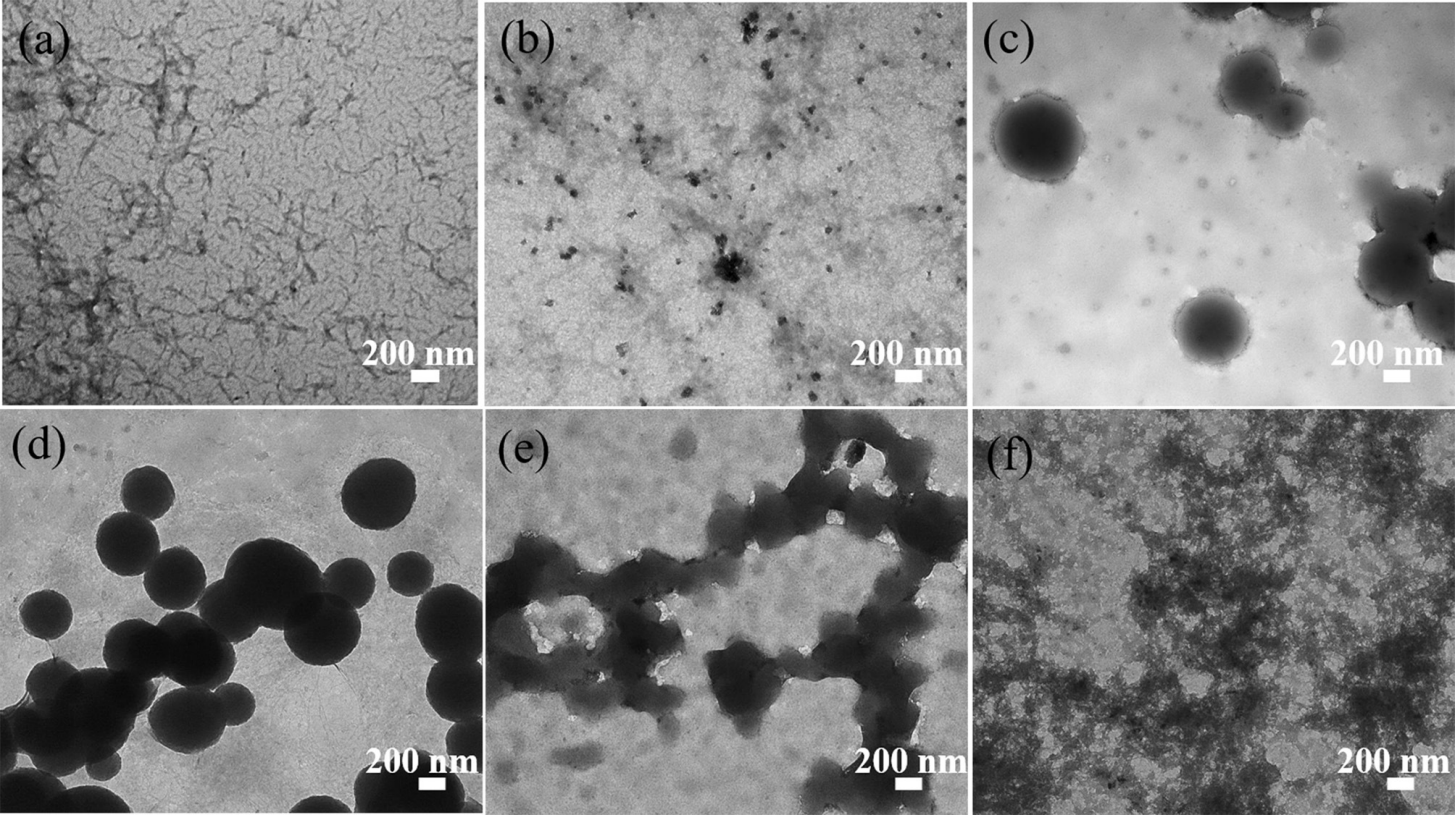
TEM images of **(a)** ANMs-C_8_H_17_-1, **(b)** ANMs-C_8_H_17_-2, **(c)** ANMs-C_8_H_17_-3, **(d)** ANMs-C_8_H_17_-4, **(e)** ANMs-C_8_H_17_-5, **(f)** ANMs-C_8_H_17_-6.

**Figure 8:**
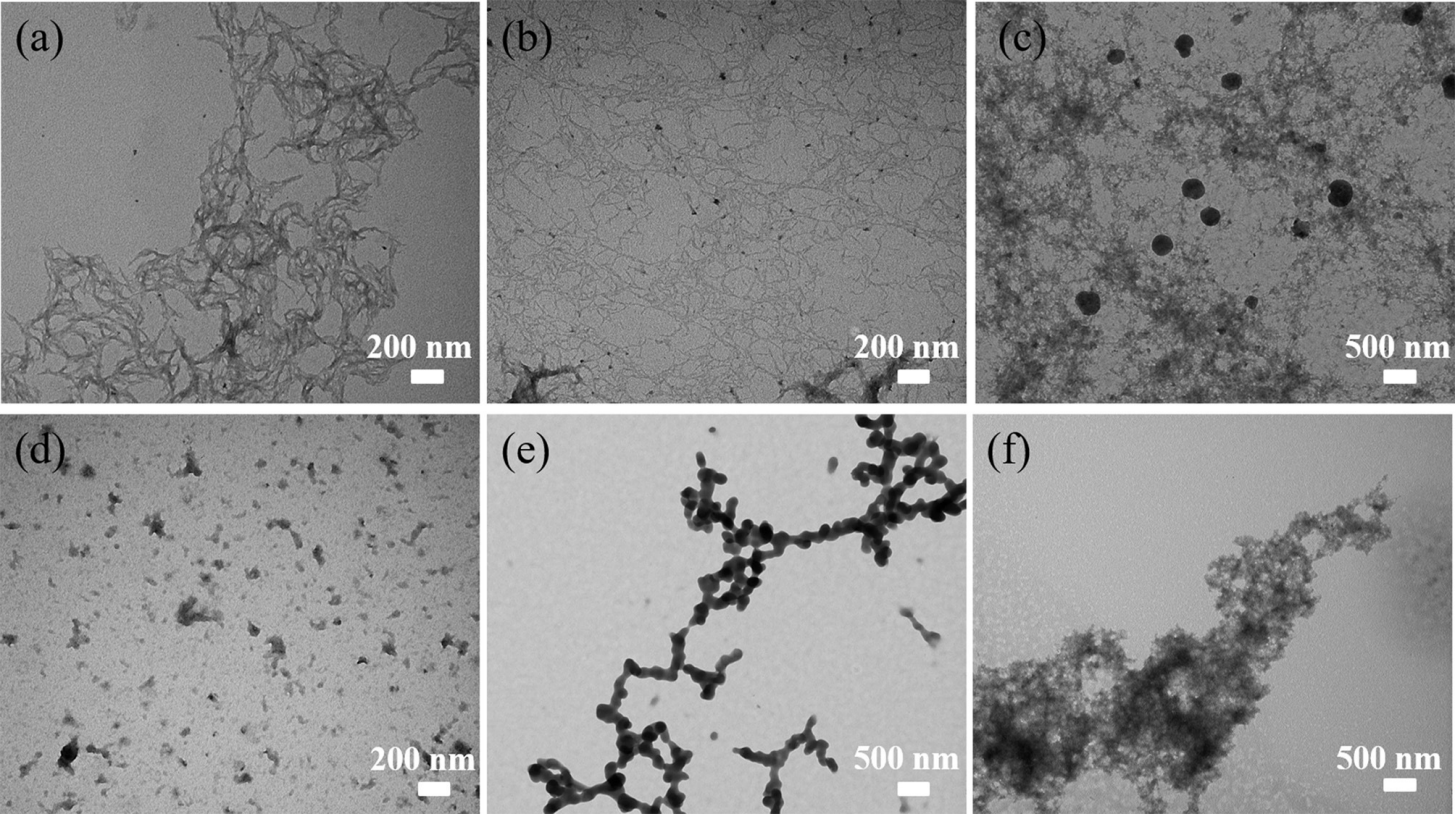
TEM images of **(a)** ANMs-C_12_H_25_-1, **(b)** ANMs-C_12_H_25_-2, **(c)** ANMs-C_12_H_25_-3, **(d)** ANMs-C_12_H_25_-4, **(e)** ANMs-C_12_H_25_-5, **(f)** ANMs-C_12_H_25_-6.

The TEM images presented in Figures 6–8 show the gradual changes in morphology with the degree of substitution for ANMs-C_2_H_5_-X, ANMs-C_8_H_17_-X, and ANMs-C_12_H_25_-X, respectively. In each of the three figures, the degree of substitution increases from (a) to (d). Concomitantly, the morphology of the resulting material changes from nanofibers to nanospheres and eventually to nanosheets. Take ANMs-C_2_H_5_-X for example. In Figures 6a,b, ANMs-C_2_H_5_-1 and ANMs-C_2_H_5_-2 exhibit in the form of short nanofibers. In Figure 6c, nanobranches are forming. In Figures 6d,e, nanospheres with the diameter of 70–350 nm are visible. In Figure 6f, nanosheets begin to appear when the degree of substitution reaches 80%.

The formation of nanospheres can be explained as follows. First, the substitution of alkyl groups at the N site of PPTA weakens or even eliminates the intermolecular interaction between PPTA molecules, which leads to the disintegration of the nanofibers (Yan et al., 2016). Second, the PPTA molecules with medium degrees of substitution (ANMs-C_2_H_5_-4 and ANMs-C_2_H_5_-5) entangle with each other and form a spherical structure which is more stable than the fibrous structure (Xu et al., 2016). The formation of nanosheets can be attributed to the breakdown of the aramid backbones and truncation of nanofibers as a result of extensive hydrolysis (Cao et al., 2013).

### Fabrication of Composite Films

**Figure 9:**
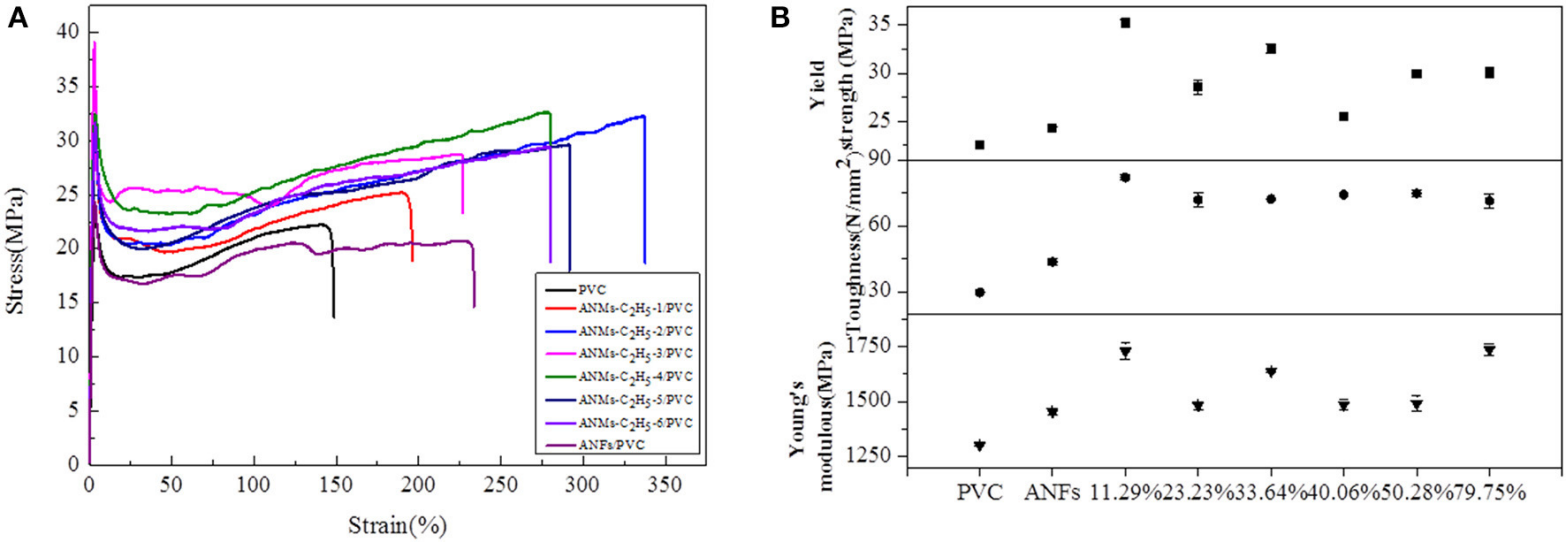
**(A)** Typical stress-strain curves, **(B)** Tensile strength, Toughness, and Young's modulus of ANMs-C_2_H_5_-X/PVC composite films with certain loading amounts of ANMs-C_2_H_5_-X.

**Figure 10:**
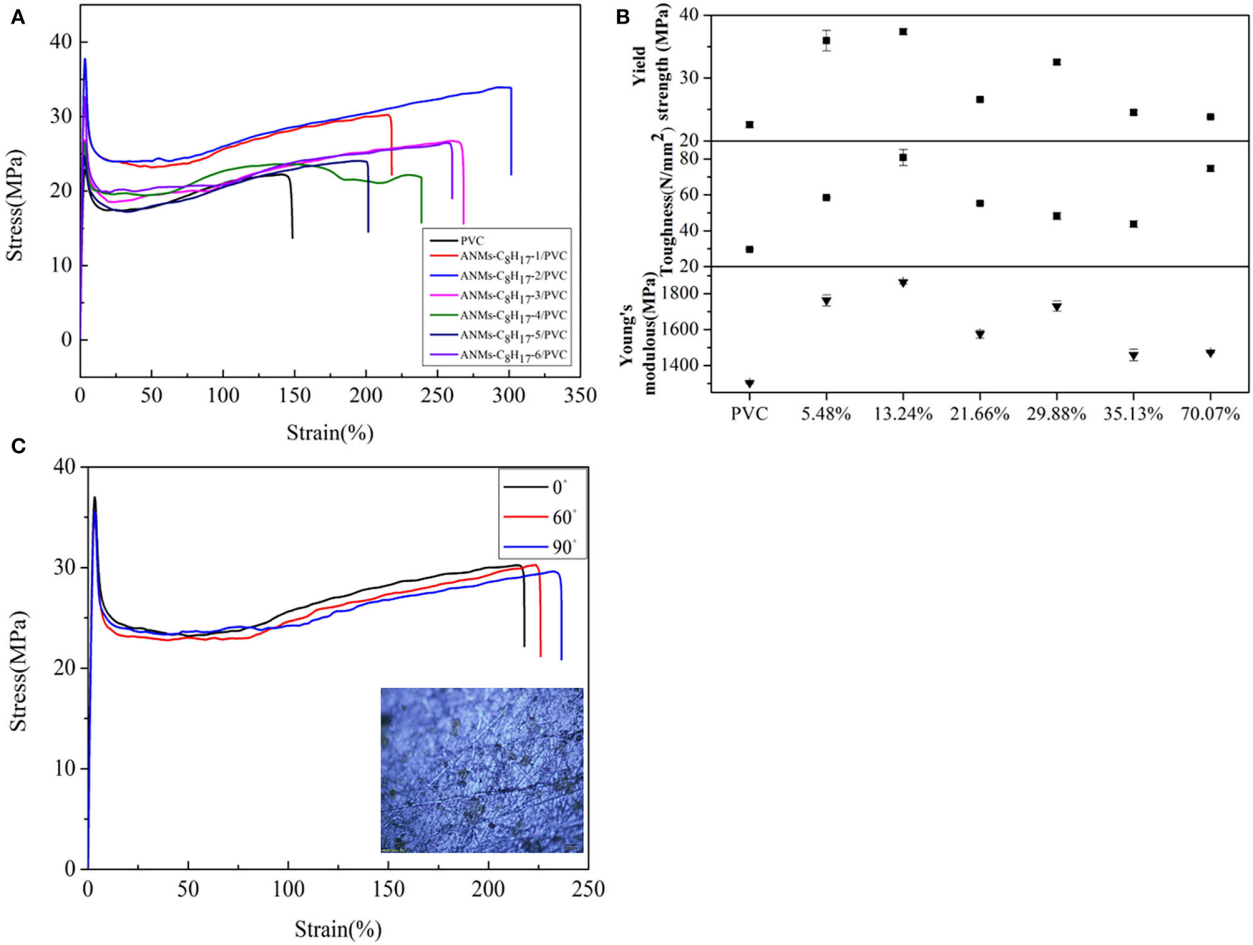
**(A)** Typical stress-strain curves, **(B)** Tensile strength, Toughness, and Young's modulus of ANMs-C_8_H_17_-X/PVC composite films with certain loading amounts of ANMs-C_8_H_17_-X. **(C)** The stress-strain curves of ANMs-C_8_H_17_-1 with cutting composite film sample in accordance with the angle of 0°, 60°, and 90°.

**Figure 11:**
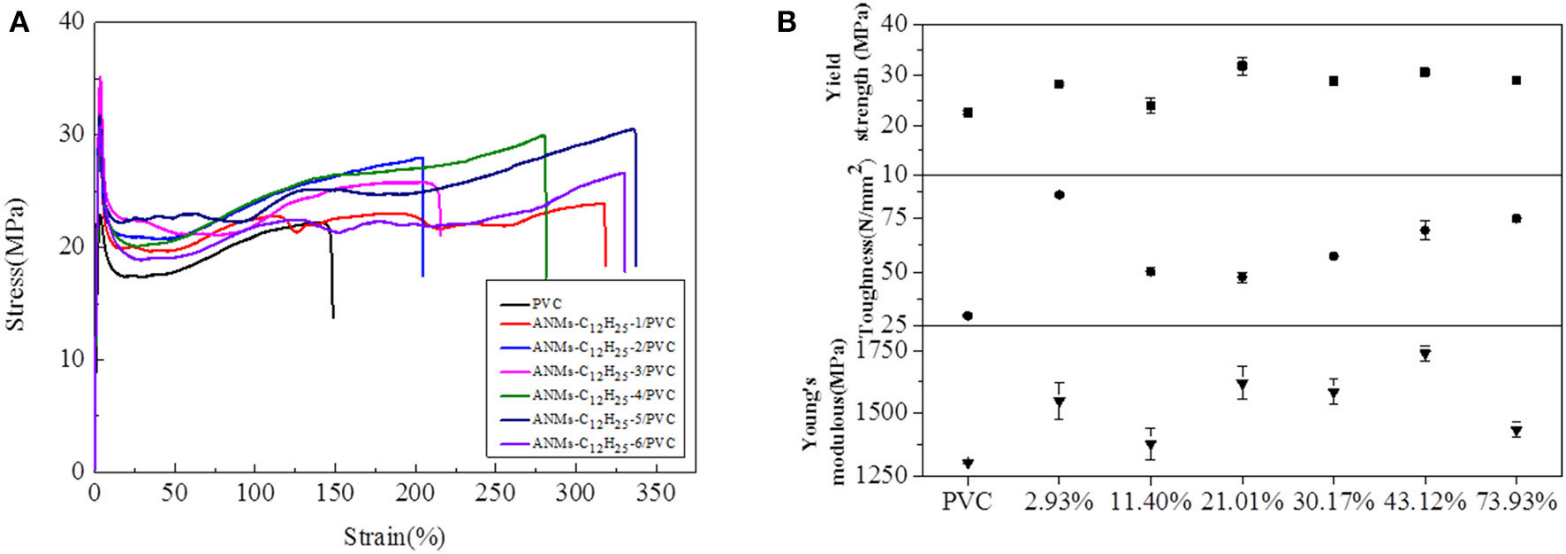
**(A)** Typical stress-strain curves, **(B)** Tensile strength, Toughness, and Young's modulus of ANMs-C_12_H_25_-X/PVC composite films with certain loading amounts of ANMs-C_12_H_25_-X.

Composite films were fabricated by dispersing R-ANMs additives in a poly(vinyl chloride) (PVC) matrix. In comparison to ANFs, the R-ANMs have better dispersibility in the polymer matrix due to the introduction of an alkyl group. The polarity similarity between R-ANMs and the PVC matrix allows for better incorporation and hence improved mechanical properties. Typical stress-strain curves as well as the tensile strength, Young's modulus, and toughness of ANMs-C_2_H_5_-X/PVC, ANMs-C_8_H_17_-X/PVC, and ANMs-C_12_H_25_-X/PVC nanocomposite films are shown in Figures 9–11, respectively.

The tensile strength, Young's modulus, and toughness for the composite films saw significant increases as compared to the PVC film without additives in all three cases. The R-ANMs additives were studied at a dosage level of 0.15 wt% for the following comparison. For the ANMs-C_2_H_5_-X/PVC films (Figure 9), the ANMs-C_2_H_5_-1/PVC film showed the maximum yield strength and maximum toughness with the additive in the form of nanofibers. The ANMs-C_2_H_5_-6/PVC film showed the maximum Young's modulus with the additive in the form of nanosheets. For the ANMs-C_8_H_17_-X/PVC films (Figure 10), the ANMs-C_8_H_17_-2/PVC film showed the highest Young's modulus, toughness, and yield strength with the additive in the form of short fibers. Moreover, in order to identify directional properties of nanofibers added film, both stress-strain curve of ANMs-C_8_H_17_-1 with cutting composite film sample in accordance with the angle of 0°, 60°, and 90° and optical microscopy images of ANMs-C_8_H_17_-1 were supplied. As shown in Figure 10C, the three stress-strain curves have similar trends. In addition, optical microscope image of ANMs-C_8_H_17_-1/PVC film with 0.15 wt% of ANMs-C_8_H_17_-1 showed a homogeneous and uniform surface (Figure 10C, inset), indicating that the additives were isotropic in the film. For the ANMs-C_12_H_25_-X/PVC films (Figure 11), the ANMs-C_12_H_25_-3/PVC film showed the highest tensile strength with the additive in the form of nanospheres. The ANMs-C_12_H_25_-1/PVC films showed the highest toughness and the ANMs-C_12_H_25_-5/PVC showed the highest Young's modulus. ANFs possess a large surface area and good mechanical properties (Yang et al., 2011), making possible the effective stress transfer from PVC to ANFs via the interface upon the introduction of tension. The strong interactions between PVC and ANMs-C_12_H_25_-X inhibit phase separation, which is beneficial to improving the tensile strength of PVC. Moreover, it is noted that the R-ANMs/PVC composite film still keep excellent toughness without the decrease of yield strength. The simultaneous increases in both tensile strength and toughness of R-ANMs/PVC films were attributed to the following: Firstly, the alkyl group grafted on the surface of ANFs could facilitate the dispersion of ANFs in the polymer matrix, which could prevent stresses concentrating at certain points. Secondly, the reformed new hydrogen bonding interactions between PVC chains and R-ANMs facilitate the stress transfer, leading to the increase of extensibility and toughness.

The maximum values for the tensile strength, toughness, and Young's modulus of the composite films are summarized in Table 2. The percent increase values as compared to the PVC film without additives are presented in the parentheses. The percent increase values were all significant, especially for toughness, which was as high as 190.24% for ANMs-C_12_H_25_-X/PVC film.

**Table 2:** Summary of the mechanical properties of ANFs and R-ANMs based polymer composite films.

**Polymer composite**	**Maximum value**			**Percent increase (%)**		
	**Tensile strength (MPa)**	**Toughness (N/mm^**2**^)**	**Young's modulus (MPa)**	**Tensile strength**	**Toughness**	**Young's modulus**
Pure PVC	22.57	29.61	1302.87	–	–	–
ANFs/PVC	24.30	43.54	1452.92	7.67	47.04	11.52
ANMs-C_2_H_5_-X/PVC	35.24	82.03	1737.16	56.14	177.03	33.33
ANMs-C_8_H_17_-X/PVC	37.37	80.77	1863.70	65.57	172.78	43.05
ANMs-C_12_H_25_-X/PVC	31.87	85.94	1740.27	41.21	190.24	33.57

### Mechanism of Mechanic Property Enhancement

The composite films were studied by TGA, and FTIR to understand the mechanism of mechanical property enhancement.

#### TGA Analysis

**Figure 12:**
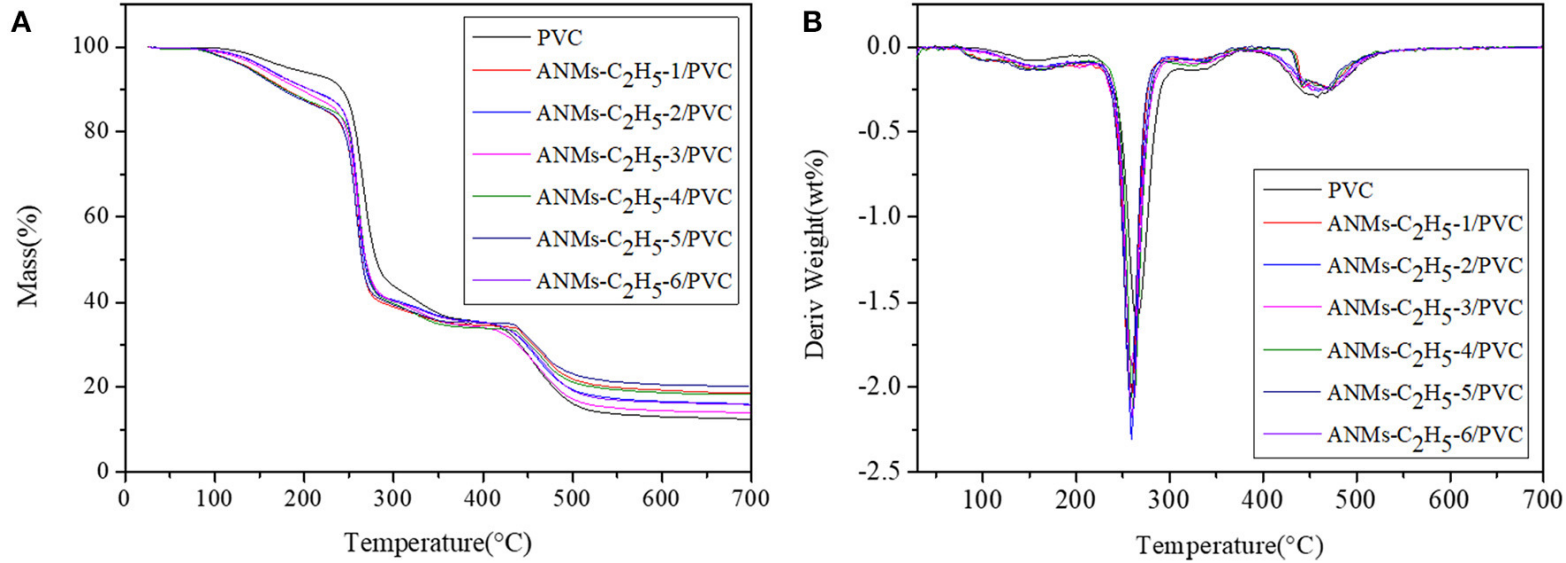
**(A)** TGA and **(B)** DTG curves of pure PVC and ANMs-C_2_H_5_-X/PVC films.

Thermogravimetric analysis (TGA) was used to characterize the thermal stability of PVC and ANMs-C_2_H_5_-X/PVC nanocomposites. The TGA and relevant derivative plots (DTG) were presented in Figure 12. A small weight loss from room temperature to 200°C was observed in all samples, which was attributed to the evaporation of the water absorbed in PVC and the residual solvent of DMSO. For pure PVC, the DTG curves showed two distinct peaks associated with two decomposition processes. The first peak from 200 to 300°C could be assigned to the decomposition of the side chain of PVC with the formation of volatile products, while the second weak peak above 350°C was mainly due to the decomposition of backbone of PVC accompanied by the formation of carbon and hydrocarbons. For ANMs-C_2_H_5_-X/PVC films, the DTG curves showed a major mass loss at 490°C, which was attributed to the decomposition of ANMs-C_2_H_5_-X. The results are consistent with similar studies of Kevlar reported in literature (O'Connor et al., 2009b; Arrieta et al., 2011). On the other hand, the addition of 0.15 wt% ANMs-C_2_H_5_-X into the PVC matrix resulted in little change in the maximum decomposition temperature of the material. This is an advantage as compared to the literature results (Yamada et al., 1986), where the mechanical property was improved but the thermal stability was negatively impacted.

#### FTIR Analysis

**Figure 13:**
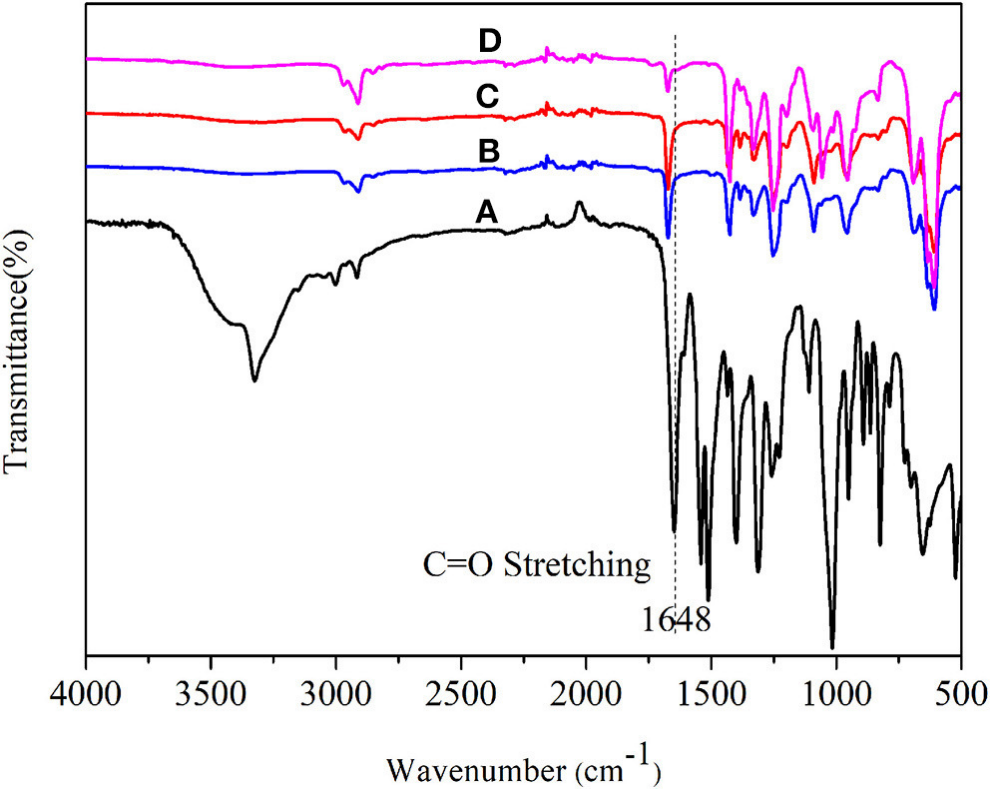
FT-IR spectra of **(A)** ANFs, **(B)** ANFs/PVC, **(C)** ANMs-C_2_H_5_-1/PVC, and **(D)** ANMs-C_2_H_5_-2/PVC composites films.

Figure 13 compares the FTIR spectra of ANFs and the composite films. The C = O stretching band at around 1,648 cm^−1^ in ANFs (Figure 13A) shifted to higher wavenumbers in ANFs/PVC (Figure 13B), ANMs-C_2_H_5_-1/PVC (Figure 13C), and ANMs-C_2_H_5_-2/PVC (Figure 13D). This can be explained by the fraction of the partial dissociation of the intermolecular hydrogen bond between N-H and C = O in ANFs (Yamada et al., 1986). This partial dissociation would be originated by the formation of another hydrogen bond between N-H of PPTA and C-Cl of PVC, and takes place on the surface of ANFs and R-ANMs formed in the composite. This again confirms that R-ANMs are finely and homogeneously dispersed in the PVC matrix.

## Conclusions

ANFs were successfully derivatized with alkyl groups by the N-substitution reaction to form R-ANMs with morphologies ranging from nanofibers to nanosheets. Alkyl groups of varied chain lengths reduced the polarity of the aramid materials, improving their miscibility and dispersibility with the PVC matrix. At the dosage level of 0.15 wt%, the R-ANMs did not have negative effect on the thermal stability of PVC as the derivatization was thought to be on the surface of the materials. The mechanical properties of the PVC films including yield strength, Young's modulus, and toughness were significantly improved with the addition of R-ANMs. R-ANMs show promises as a performance-enhancing additive for non-polar or weakly polar polymers.

## Data Availability Statement

All datasets generated for this study are included in the article/supplementary material.

## Author Contributions

This work was completed by cooperation of all authors. CD carried out experiments and wrote the manuscript. CS and RQ designed experiments, analyzed results, and revised the manuscript. PG and YY carried out performance-enhancing experiments of the PVC films. CJ, YZ, and YW characterized and analyzed experimental results.

### Conflict of Interest

The authors declare that the research was conducted in the absence of any commercial or financial relationships that could be construed as a potential conflict of interest.
